# Competent and compassionate, but not leading? A cross-sectional study of nursing’s brand image in the German public

**DOI:** 10.1186/s12912-026-04683-z

**Published:** 2026-04-28

**Authors:** Clemens Koob

**Affiliations:** Faculty of Healthcare and Nursing, Catholic University of Applied Sciences Munich, Preysingstraße 95, 81667 Munich, Germany

**Keywords:** Brand image of nursing, Nursing brand image scale, Germany, Public perceptions

## Abstract

**Background:**

Nursing’s public image influences workforce sustainability and broader professional and care-related outcomes. While international research has advanced understanding of nursing’s public image, population-based evidence for Germany remains scarce and methodologically limited. Drawing on brand image theory, this study assessed nursing’s brand image in the German public and examined heterogeneity across population subgroups, extending beyond sociodemographic factors to include lifestyle and personality characteristics.

**Methods:**

This descriptive study used data from a cross-sectional online survey of the German public with quotas on age, gender, education, and region to approximate national distributions (*N* = 950; aged 16–65), conducted in summer 2025. Nursing’s brand image was measured using the validated Nursing Brand Image Scale for the Public, German version (NBIS-P-G), comprising four dimensions capturing competence, compassion, leadership, and professional identity (1–10 scale). Descriptive statistics and repeated-measures ANOVAs were used to compare subscale means and item endorsements. Group differences across sociodemographic, lifestyle, and personality characteristics were examined using Welch’s t-tests and one-way ANOVAs. Benjamini-Hochberg corrections were applied across all families of tests to control the false discovery rate.

**Results:**

Nursing’s brand image was primarily characterized by Professional Competence and Expertise (M = 7.18) and Patient-Centered Care and Compassion (M = 7.13), both at comparable, moderately favorable levels, exceeding ratings for Leadership and Influence (M = 6.60). Endorsement of Professional Identity Challenges (M = 6.26; higher scores indicate stronger challenges) indicated image constraints. Item-level analyses showed comparatively weaker endorsement of items reflecting academic and scientific competencies and patient advocacy, as well as authority and decision-making within the leadership dimension. Sociodemographic differences were minimal. In contrast, lifestyle-related variation was observed across all dimensions, with medium-to-large effects for leadership perceptions and identity challenges, and personality-segment differences emerged for competence and care, with higher Stability associated with more favorable perceptions.

**Conclusions:**

The German public perceives nursing as competent and compassionate, yet leadership, influence, and professional distinctiveness are less strongly recognized. Lifestyle and personality characteristics represent meaningful sources of heterogeneity, suggesting branding initiatives may benefit from psychographic segmentation. These findings provide an empirical foundation for strengthening nursing’s brand image in Germany and establish a baseline for evaluating future initiatives.

**Supplementary Information:**

The online version contains supplementary material available at 10.1186/s12912-026-04683-z.

## Background

The global nursing shortage represents a key dimension of the health workforce crisis [1–3], with nursing’s public image playing a crucial role for workforce sustainability. Public perceptions influence career choice, with attributes such as prestige, role clarity, and societal value significantly associated with entry into nursing [4–15]. A contemporary, professional image emphasizing competence, responsibility, and scope of practice can support recruitment, while perceptions of limited autonomy, traditional gender roles, or lacking career perspectives may deter candidates [14, 16]. Recent research further indicates that a positive nursing image fosters nursing students’ career preparation behaviors critical for successful transition into professional practice [17].

Beyond recruitment, nursing’s public image affects professional identity and retention. How nurses believe they are viewed by society influences their self-concept, professional pride, and resilience [14, 18, 19]. An unfavorable image contributes to attrition, while positive perceptions motivate retention [20]. Recent reviews have further highlighted that the influence of nursing’s public image extends to individual-level outcomes such as job satisfaction, performance, and work-life quality, as well as organizational and public-level outcomes including interprofessional collaboration, quality of care, and population health [14, 16].

Research on nursing’s public image has generated substantial evidence over past decades [14, 16, 21, 22], with recent syntheses clarifying the construct’s evolution and key elements. Drawing on systematic review of international literature, Duan et al. [14] delineated nursing’s public image as comprising intrinsic (professional spirit, knowledge, skills) and extrinsic dimensions (appearance, language, behavior in professional and extra-professional contexts).

Building on this conceptual work and converging international evidence [16, 21, 23–25], several recurrent themes emerge regarding how nursing is perceived publicly and which factors contribute to these perceptions. First, public perception emphasizes caring and empathy, with attributes such as friendliness, warmth, and compassion outweighing associations with technicity, autonomy, leadership, or research [24–26]. Second, nursing continues to be viewed as a low-status, subordinate profession with limited autonomy and lower educational levels [14, 16, 23, 24]. Third, despite evolving perceptions [27], nursing remains largely viewed as a feminine profession, with caring regarded as innate to women and stereotypes questioning male nurses’ masculinity and competence [14, 23, 28]. Fourth, broader stereotypes of nurses as physicians’ “handmaidens”, empathetic “angels”, “naughty nurses”, or pandemic-era “heroes” are engrained into public image [14, 29–33]. Media representation [16, 23, 34–37] and generative AI [33] reinforce such gendered or stereotyped images. Fifth, visual cues such as uniforms often lack clear differentiation from other staff, diminishing perceptions of nursing as a distinct profession [14, 16]. Sixth, the public often lacks accurate understanding of nursing realities, including work nature, autonomy, role diversity, career opportunities, and remuneration [14, 16, 21, 23, 25–27, 38, 39]. Seventh, major health crises such as the COVID-19 pandemic can – at least temporarily – enhance public recognition of nurses’ capabilities and social contribution, demonstrating the image’s responsiveness to contextual change [14, 28–30, 40–42]. Collectively, these themes suggest that international research portrays nursing’s public image as characterized by caring and relational qualities alongside limitations in perceived autonomy, professional status, and distinctiveness.

Notably, while cross-national differences in nursing’s public image are well-documented [24, 26], findings regarding variation across population subgroups remain inconclusive. Age-related patterns show contradictory results: some studies suggest older adults hold more traditional views [43, 44] or attribute higher prestige to nursing [24], while others found no significant age effects [26]. Evidence on gender effects is similarly mixed, with some studies reporting no differences [24, 26] and others finding men less likely to hold positive nursing images [39]. Educational attainment was positively correlated with nursing perceptions in one study [26]. Research beyond sociodemographic variables is scarce, with one study observing that generational identity shapes public perception of nursing [45].

Taken together, international research has substantially advanced understanding of nursing’s public image, with perceptions varying across societies [24, 26], shaped by historical, cultural, political, economic, and media contexts as well as healthcare systems and nursing education characteristics [14]. Examining perceptions within specific national contexts therefore remains essential.

With the highest number of practicing nurses among EU countries [46] and a supply-oriented healthcare system (social insurance-based, with high provider supply and a high degree of physicians’ autonomy [47]), Germany represents a particularly relevant setting. In recent years, nursing has received substantial public attention through the COVID-19 pandemic [48, 49], political reforms [e.g., 50–52], workforce shortages [53], nurse influencer activity [54], and related media coverage [55, 56], with public institutions investing in campaigns to enhance the profession’s attractiveness [57, 58]. Notwithstanding this attention, recent and methodologically robust insights into how the German public perceives nursing remain limited. Extant publications compile secondary data [55], are limited to occupational prestige rankings [59], focus on media discourse and content analyses [48, 49, 60], or comprise ad-hoc surveys among specific subgroups such as school students [61]. Labonte [48] noted that nurses continue to struggle for social recognition, with nursing’s image remaining shaped by historical remnants of a feminine, church-bound charitable profession. Consistent with this, studies among German adolescents found nursing primarily characterized by industriousness (diligence, trustworthiness, physical fitness) and social qualities (sociability, dexterity, altruism), while education, intelligence, wealth, and prestige were attributed to nurses to a considerably lesser extent [61]. General population surveys, however, present contrasting findings: recent panel data found general and geriatric nurses ranking second and third among 31 occupations in terms of occupational prestige [59]. In sum, extant research provides only a fragmented picture of nursing’s image among the broader German population.

Beyond substantive gaps in the German evidence base, methodological limitations constrain research on nursing’s public image internationally and within Germany. Many studies have relied on non-validated self-developed questionnaires [24, 27, 29, 38, 61], non-validated translations [7, 28], or measures validated in non-public samples [8, 31]. Only more recently have studies reported results based on instruments with established psychometric properties – for instance, Godino et al. [45] in Italy used the Scale for the Image of the Nursing Profession (I-SINP), and Zhou et al. [26] in the United States and China applied the Nursing Brand Image Scale for the Public (NBIS-P). To our knowledge, no validated instrument has yet been applied to assess nursing’s public image in Germany.

Within the limited international landscape of validated instruments [62], the NBIS-P is distinctive in being grounded in brand image theory from marketing, conceptualizing nursing as a professional brand [63, 64]. In this perspective, nursing brand image refers to the collective, socially constructed perception of the profession, comprising functional, symbolic, and experiential associations [65–67]. This branding perspective enables multidimensional analysis of how nursing is cognitively represented, emotionally evaluated, and socially positioned, extending beyond global attitudes or prestige ratings. Moreover, brand image research recognizes that perceptions may vary across population segments defined not only by sociodemographic characteristics but also by psychographic factors such as personality traits, as well as by lifestyle and (sub)cultural orientations [68, 69], given their various regulative functions [70, 71]. The NBIS-P has recently been adapted and validated for the German public (NBIS-P-G) [72], providing a theoretically grounded and psychometrically robust tool.

Accordingly, brand image theory [65, 67] constitutes the core theoretical foundation of this study, guiding conceptualization, measurement, and interpretation of nursing’s public image as a brand image. This adds analytical value beyond traditional image research by conceptualizing perceptions as multidimensional networks of positive and negative associations in peoples’ minds and by acknowledging that different population segments may construct distinct brand images [67, 69]. Related constructs commonly used in nursing research tend to be less theoretically specified: “public perception” typically remains rather vaguely defined as an expression of beliefs and sentiments [22, 73], “public image” encompasses diverse definitions without consistent theoretical grounding [14], and “occupational prestige“ captures only a unidimensional evaluative ranking of occupational desirability [74]. Moreover, branding theory explicitly acknowledges the profession’s positioning relative to others in a brand ecosystem and frames brand image as a strategically relevant and potentially manageable asset [65, 67]. In line with the interdisciplinary nature of branding research [75], we drew on complementary interpretative lenses from sociology and psychology to interpret specific patterns in how nursing is perceived and how these perceptions vary across population groups.

Against this background, this study aimed to examine the nursing brand image in the German public using the recently validated NBIS-P-G. Specifically, the study aimed (1) to provide a current, population-based assessment of how nursing is perceived within the German public, and (2) to explore differences in image perceptions across population subgroups, extending beyond sociodemographic factors to include lifestyle and personality characteristics. This responds to mixed international findings regarding sociodemographic variation and the scarcity of evidence on the role of psychographic factors in the perception of the nursing profession. Branding research suggests that lifestyle and personality characteristics exert regulative functions for perceptions and evaluative judgments [68–71], warranting their examination in the context of nursing’s brand image.

## Methods

### Study design

This manuscript reports a secondary, descriptive analysis of the dataset used to validate the Nursing Brand Image Scale for the Public, German version (NBIS-P-G) [72], derived from a cross-sectional online survey conducted in summer 2025 using EFS Survey v24.2. No new data were collected for the present analyses.

The survey included the NBIS-P-G as the primary measure [72]. Additional measurement instruments were incorporated to enable subgroup comparisons across sociodemographic, lifestyle, and personality characteristics. All questionnaire items were presented in German.

The questionnaire was pretested with 41 university students (bachelor’s/master’s level) to assess comprehensibility and survey flow; feedback led to minor wording and layout refinements.

Although the present manuscript uses the same dataset as the NBIS-P-G validation study [72], the psychometric results from the validation analyses were not used to select, modify, or tune the descriptive and comparative analyses reported here.

The study protocol was approved by the Interdisciplinary Research Ethics Committee, Catholic University of Applied Sciences Munich (approval 2024/N48-2). Participation was voluntary, and all respondents provided informed consent prior to starting the survey. Data collection complied with the EU General Data Protection Regulation (GDPR). Reporting follows STROBE for cross-sectional studies [76] and STROSA-2 for secondary data analyses [77].

### Participants and sampling

Eligible participants were residents of Germany aged 16–65 years. We recruited participants through an online access panel to enable efficient sampling, as commonly done in research [78, 79]. Cint was selected as provider because they operate one of the largest German panels and adhere to a transparent sampling process consistent with the ESOMAR framework for evaluating online sampling services [80]. Non-interlocking quotas on age, gender, education, and region of residence were used to approximate national composition of the sample. Eligibility was verified at entry via screening items on these variables; quotas were pre-specified from national statistics [81–83] and monitored in real time. No post-hoc weighting was applied, as quotas served as the representativeness mechanism.

### Measures

#### Nursing brand image

Nursing’s brand image was assessed with the Nursing Brand Image Scale-Public, German version (NBIS-P-G) [72], a validated German-language instrument for assessing public perceptions of nursing. The original NBIS was developed in the United States to measure nurses’ self-perceptions [63] and later adapted for public perceptions [26]. The recent German validation study confirmed a four-factor structure with good psychometric properties [72]. Internal consistency in the validation sample, which also constitutes the analytic sample for the present study, ranged from acceptable to excellent (*Professional Competence and Expertise*: α = 0.95, ω = 0.95; *Patient-Centered Care and Compassion*: α = 0.93, ω = 0.94; *Leadership and Influence*: α = 0.88, ω = 0.91; *Professional Identity Challenges*: α = 0.74, ω = 0.74).

The NBIS-P-G comprises 36 descriptors across four subscales: *Professional Competence and Expertise* (16 items; e.g., “fachkundig” [skilled], “evidenzbasierte Praxis” [evidence-based practice], “kompetent” [competent]), *Patient-Centered Care and Compassion* (9 items; e.g., “fürsorglich/mitfühlend” [caring/compassionate], “empathisch” [empathetic], “patientenorientiert” [patient centered/focused]), *Leadership and Influence* (8 items; e.g., “Führungspersonen” [leaders], “eigenständig” [autonomous], “einflussreich” [influential]), and *Professional Identity Challenges* (3 items: “untergeordnet/dienend” [subservient], “schwer von anderen zu unterscheiden” [hard to identify from others], “Berufskleidung vermittelt keine Professionalität” [attire does not reflect professionalism]).

Respondents rated the extent to which each descriptor applies to the nursing profession on a 10-point scale (1 = does not apply at all, 10 = perfectly applies). Subscale scores were computed as the mean of items within each domain; higher scores indicate stronger endorsement. A total score was not computed, consistent with the scale’s multidimensional conceptualization [72]. Item order was randomized for each respondent to mitigate order effects. The complete item list with German and English versions is provided in Additional file 1 (German version: [72]; original English version: [63]).

#### Other measures

##### Socio-demographics

Socio-demographics were collected following national statistics [81] and German data collection recommendations [84]. Age was recorded in years and recoded into four categories: 16–29, 30–39, 40–49, 50–65. Gender used a binary classification (female, male) as specified in official German statistics for quota setting. Education level followed German microcensus classification: low (compulsory/lower secondary, grade 9 completion), medium (lower secondary, grade 10 completion), and high (university entrance qualification or degree). Region of residence was recorded by federal state and grouped into four macro-regions (North, West, East, South) [85].

##### Lifestyles

Lifestyles were classified using Otte’s theoretically informed typology operationalized via the Münster Life Conduct Typology (MLCT) [86, 87]. Two dimensions – material level (economic and cultural capital) and modernity / biographical perspective (modernity of life conduct and values) – were measured with 7 items each from the 14-item diagnostic statement scale (1 = does not apply at all; 4 = applies completely) [88]. Dimension scores were item means. For subgroup analyses, participants were assigned to five lifestyle types [89] using published norms [88] and author-provided syntax (Stelzer, personal communication; used with permission): Social Elite (high capital, traditional to partially modern; leadership and achievement orientation, refined lifestyle), Traditionalists (low-to-medium capital, traditional; conventional values, modest lifestyle), Middle Class (medium capital, partially modern; status-conscious and achievement-oriented, mainstream consumption), Avantgarde (medium-to-high capital, modern; progressive, high adaptability and innovation), and Underprivileged-Modernized (low capital, partially modern to modern; experience-oriented consumption, limited resources).

##### Personality

We used the Big Five model [71, 90] – Openness, Conscientiousness, Extraversion, Agreeableness, and Neuroticism – and its higher-order metatraits: Stability (representing stable motivational, social, and emotional functioning) and Plasticity (reflecting tendencies to explore and engage flexibly with novelty) [91, 92]. Personality was measured with the validated German BFI-10 [93] on a 5-point response scale. Following Dunkel et al. [94], metatraits were derived as metric composites from z-standardized trait scores (detailed formulas in Additional file 2). For subgroup analyses, respondents were assigned to five personality segments using ± 0.5 SD thresholds on both metatraits [95]: High Stability-High Plasticity, High Stability-Low Plasticity, Low Stability-High Plasticity, Low Stability-Low Plasticity, and Others (respondents not in one of the four high/low quadrants).

### Bias and data quality

To mitigate data quality risks related to online panel sampling, we implemented multiple safeguards [79, 96]. Potential economic self-selection bias [78] was addressed by controlling the sample’s socioeconomic composition via quotas and relying on standard, panel-administered incentives [96]. Provider fraud/identity vetting [97] and the eligibility screeners ensured an eligible sample. At survey entry, respondents affirmed a careful-responding commitment [98]; non-committers were excluded. We embedded attentiveness checks and excluded any respondent who failed them [99]. We computed a longstring index on the NBIS-P-G (maximum number of identical consecutive responses); respondents with ≥ 50% identical responses were classified as careless/insufficient-effort and excluded [99]. To minimize item nonresponse, mandatory answering was used [100].

To address common method bias in self-reports, we applied procedural remedies [101]: psychological separation of constructs via labeled sections and neutral transitions, varied response formats and verbal anchors across instruments, randomized item order within multi-item instruments, and concise questionnaire design.

### Statistical analyses

#### Data preparation

Procedures matched those used in the NBIS-P-G validation study [72]. Respondents were excluded based on the eligibility, commitment, attention, and longstring criteria described above. Mandatory answering prevented missing values; no imputation or listwise deletion was required.

#### Effect size reporting

Effect sizes are reported with 95% confidence intervals (CIs). For omnibus ANOVAs (within-subjects and between-groups), we report partial eta-squared $(\:{{\eta\:}_{p}}^{2})$. Partial eta-squared was selected given its predominant use in ANOVA designs [102, 103]. Although it is known to be positively biased [104, 105], it was also chosen because less biased alternatives such as omega-squared face a lack of consensus on which formulas to use for calculation, especially for designs including within-subjects factors, as well as a lack of research into CIs for such designs [103], limiting their applicability in the present study. Partial eta-squared values should accordingly be interpreted cautiously. For paired comparisons, we report Cohen’s d_z_; for independent comparisons, we report Cohen’s d calculated with the mean of the two group SDs. Conventional benchmarks (small / medium / large) are provided for orientation $(\:{{\eta\:}_{p}}^{2}$: 0.01 / 0.06 / 0.14; d_z_, d: 0.20 / 0.50 / 0.80) [106]. Classification is determined with reference to the 95% CI relative to these benchmarks.

#### Brand image and subscale mean differences

To describe how the German public rates the brand image of the nursing profession, the means and standard deviations of the four NBIS-P-G subscales were calculated. To test whether the subscales differed in their mean scores, we conducted a repeated measures ANOVA with subscale (1–4) as the within-subjects factor. Mauchly’s test assessed sphericity; Greenhouse-Geisser correction was applied to degrees of freedom and p-values when violated (α = 0.05).

When the omnibus test indicated significant differences between subscales, post-hoc pairwise comparisons were conducted using paired t-tests. P-values were adjusted using the Benjamini-Hochberg correction to control the false discovery rate across the six pairwise comparisons.

#### Item-level analysis within subscales

For each subscale, we examined item-endorsement patterns using repeated measures ANOVA supplemented by graphical exploration.

##### Omnibus tests

A repeated measures ANOVA was conducted for each subscale separately, with item as the within-subjects factor, to assess whether items within that subscale exhibited significant variation in endorsement. Sphericity was assessed and corrected as described above. No correction for multiple comparisons was applied across the four subscales, as each analysis addressed a distinct question regarding item heterogeneity within a specific domain.

##### Patterns of item means

To support interpretation, we computed means and difference-adjusted 95% Cousineau-Morey CIs [107] for all items within each subscale. These CIs are conceptualized for within-subjects comparisons; non-overlap corresponds to a 95% CI for the difference between means that excludes zero [107]. Item means with their difference-adjusted CIs are presented graphically. Detailed descriptive statistics are reported for selected items showing notably high or low endorsement relative to others within the same subscale. This approach allows interpretation of item-level patterns without requiring excessive formal pairwise comparisons (ranging from 3 comparisons for subscale 4 to 120 for subscale 1), consistent with recommendations by Baguley [107].

#### Group comparisons of subscale scores

We examined differences in subscale scores across demographic (gender, age, education, region), socio-cultural (lifestyles), and psychological (personality metatraits) grouping variables.

##### Two-group comparisons

For gender, Welch’s t-tests were conducted for each subscale, as recommended for their robustness to unequal variances [108, 109]. As these comparisons constitute a family of related tests, p-values were adjusted using the Benjamini-Hochberg procedure to control the false discovery rate.

##### Multi-group comparisons

For grouping variables with three or more levels, Welch’s one-way ANOVA was conducted for each subscale [110, 111]. P-values were adjusted across the four omnibus tests (one per subscale) for each grouping variable using the Benjamini-Hochberg procedure to control the false discovery rate. When significant differences were indicated (adjusted *p* < 0.05), post-hoc pairwise comparisons were conducted using the Games-Howell test, which adjusts for multiple comparisons and does not assume equal variances.

#### Software

Analyses were conducted in R (v4.3.2) and RStudio (v2023.06.1) using packages careless (v1.2.2; longstring checks), afex (v1.4-1; repeated measures ANOVAs), emmeans (v1.11.2; within-subjects pairwise comparisons), stats (v4.3.2; Welch’s t-tests and one-way ANOVAs), and effectsize (v1.0.1; effect size indices). Difference-adjusted Cousineau-Morey CIs were computed using the custom function from Baguley [107]. Statistical significance was set at α = 0.05 for all tests, with multiplicity adjustment as described above.

### Effect-size sensitivity analysis

Because sample size was fixed by the NBIS-P-G validation dataset (*N* = 950 [72]), no new a priori power analysis was conducted. Instead, following methodological guidance [112], effect-size sensitivity analyses were performed to determine the minimum detectable effects for key analyses at α = 0.05 and 80% power. Sensitivity analyses were conducted using G*Power (v3.1), with assumptions tailored to the respective statistical tests (repeated measures ANOVAs, paired and independent t-tests, and Welch’s one-way ANOVAs). Detailed assumptions and sensitivity parameters are provided in Additional file 3.

Minimum detectable effects are reported alongside the results of the main analyses using the same effect-size metrics. Consistent with current recommendations [112], observed power is not reported.

## Results

### Participant characteristics

The analytic sample consisted of 950 participants, corresponding to the final dataset used in the NBIS-P-G validation study [72]. This sample resulted after exclusions for ineligibility (*n* = 28), failed commitment or attention checks (*n* = 164), and longstring responding (*n* = 103).

The analytic sample was balanced by gender (51.7% female, 48.3% male; Table 1). The 50–65 age group constituted the largest proportion (31.4%). The highest proportion of respondents held higher education degrees (43.3%). Geographically, western Germany (38.7%) and southern Germany (24.6%) were most represented. Compared with national benchmarks, the sample matched the target population across measured characteristics; the largest absolute deviation was 5.1 percentage points (South underrepresented: 24.6% vs. 29.7%) [81–83].

**Table 1:** Sample characteristics and comparison with target population

	Sample		Target population
	*n*	%	%
**Gender**			
Female	491	51.7	49.3
Male	459	48.3	50.7
**Age (years)**			
16–29	243	25.6	24.9
30–39	217	22.8	20.8
40–49	192	20.2	19.5
50–65	298	31.4	34.8
**Education level**			
Low	218	22.9	25.6
Mid	321	33.8	32.3
High	411	43.3	42.1
**Region**			
North	155	16.3	16.2
West	368	38.7	35.4
East	193	20.3	18.6
South	234	24.6	29.7

Note: *N* = 950; education level per microcensus: low = compulsory / lower secondary (grade 9), mid = lower secondary (grade 10), high = university entrance or degree

### Brand image and subscale mean differences

To examine how the German public rates the nursing brand image, the four NBIS-P-G subscale means were compared using a repeated measures ANOVA. Mauchly’s test indicated a violation of sphericity, W = 0.43, *p* < 0.001. Therefore, Greenhouse-Geisser corrections were applied ($\:\epsilon\:$ = 0.64). The repeated measures ANOVA revealed significant differences between subscale means, F(1.93, 1827.80) = 155.47, *p* < 0.001. Partial eta-squared ($\:{{\eta\:}_{p}}^{2})$ was 0.14 (95% CI [0.12, 0.16]), large by point estimate with the CI spanning the medium-large boundary. The minimum detectable effect was $\:{{\eta\:}_{p}}^{2}$ = 0.002.

Pairwise comparisons (paired t-tests, BH-adjusted p; Table 2) showed no difference between *Professional Competence and Expertise* (M = 7.18, SD = 1.57) and *Patient-Centered Care and Compassion* (M = 7.13, SD = 1.73), p_BH_ = 0.176, d_z_ = 0.04 (95% CI [-0.02, 0.11]).

Both *Professional Competence and Expertise* and *Patient-Centered Care and Compassion* were rated higher than *Leadership and Influence* (M = 6.60, SD = 1.64), both p_BH_ < 0.001. Effects were small-to-medium for *Professional Competence and Expertise* vs. *Leadership and Influence* (d_z_ = 0.52, 95% CI [0.46, 0.59]) and small for *Patient-Centered Care and Compassion* vs. *Leadership and Influence* (d_z_ = 0.43, 95% CI [0.36, 0.49]).

*Professional Competence and Expertise* and *Patient-Centered Care and Compassion* were also rated higher than *Professional Identity Challenges* (M = 6.26, SD = 1.96), both p_BH_ < 0.001. Effects were small-to-medium (d_z_ = 0.46, 95% CI [0.39, 0.52] and d_z_ = 0.45, 95% CI [0.39, 0.52], respectively).

Finally, *Leadership and Influence* was rated higher than *Professional Identity Challenges*, p_BH_ < 0.001, with a trivial-to-small effect (d_z_ = 0.20, 95% CI [0.14, 0.27]).

The minimum detectable effect was d_z_ = 0.113.

**Table 2:** Pairwise comparisons among NBIS-P-G subscales (paired t-tests, BH-adjusted p)

Contrast	t(df)	*p*_BH_	d_z_	95% CI (d_z_)
Professional Competence and Expertise vs. Patient-Centered Care and Compassion	1.35	0.176	0.04	[-0.02, 0.11]
Professional Competence and Expertise vs. Leadership and Influence	16.16	< 0.001	0.52	[0.46, 0.59]
Professional Competence and Expertise vs. Professional Identity Challenges	14.10	< 0.001	0.46	[0.39, 0.52]
Patient-Centered Care and Compassion vs. Leadership and Influence	13.14	< 0.001	0.43	[0.36, 0.49]
Patient-Centered Care and Compassion vs. Professional Identity Challenges	13.97	< 0.001	0.45	[0.39, 0.52]
Leadership and Influence vs. Professional Identity Challenges	6.25	< 0.001	0.20	[0.14, 0.27]

Note: *N* = 950; BH = Benjamini-Hochberg adjustment across six contrasts

### Item-level analysis within subscales

Following the subscale-level comparisons, item-level endorsement within each subscale was examined using repeated measures ANOVAs and difference-adjusted Cousineau-Morey CIs.

#### Professional competence and expertise

Mauchly’s test indicated a violation of sphericity, W = 0.59, *p* < 0.001. Therefore, Greenhouse-Geisser corrections were applied ($\:\epsilon\:$ = 0.93). The repeated measures ANOVA showed that item means differed, F(13.90, 13188.73) = 21.47, *p* < 0.001. Partial eta-squared ($\:{{\eta\:}_{p}}^{2})$ was 0.02 (95% CI [0.02, 0.03]), a small omnibus effect. The minimum detectable effect was $\:{{\eta\:}_{p}}^{2}$ = 0.001.

**Fig. 1:**
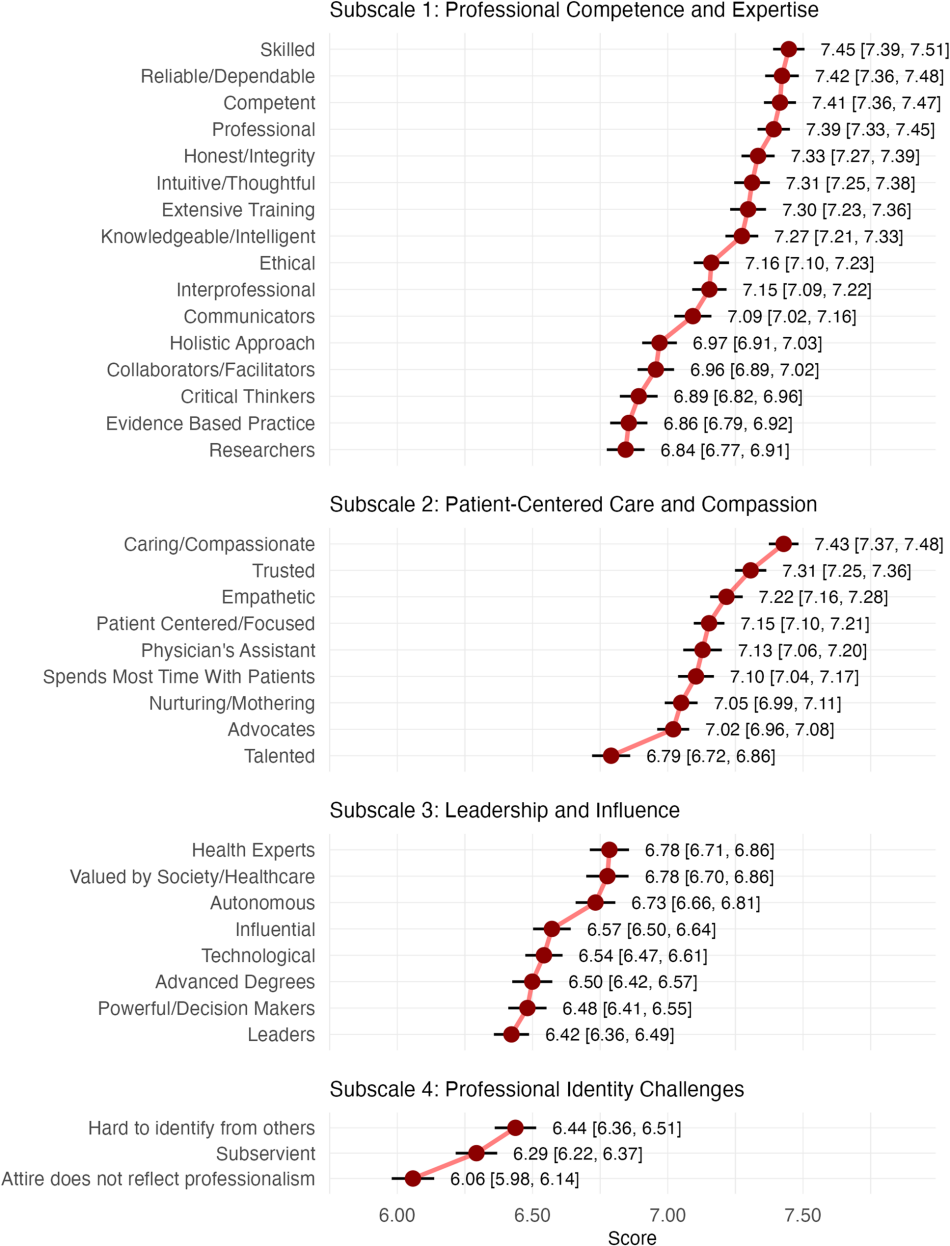
Subscale item means with difference-adjusted 95% Cousineau-Morey CIs. Note: *N* = 950. Points show item means; horizontal bars are difference-adjusted within-subjects 95% Cousineau-Morey CIs; within each subscale, items are ordered from highest to lowest mean (top to bottom); the x-axis is truncated to aid readability; exact means and CIs are printed to the right of each item; omnibus RM-ANOVA summaries are reported in the text

Item means ranged from M = 6.84 (95% CI_CM_ [6.77, 6.91]) to M = 7.45 (95% CI_CM_ [7.39, 7.51]); 11 of 16 items (69%) had point estimates $\:\ge\:$ 7.00, placing them in the upper tercile of the 10-point scale. The most strongly endorsed items were “Skilled” (M = 7.45, 95% CI_CM_ [7.39, 7.51]), “Reliable/Dependable” (M = 7.42, 95% CI_CM_ [7.36, 7.48]), “Competent” (M = 7.41, 95% CI_CM_ [7.36, 7.47]), and “Professional” (M = 7.39, 95% CI_CM_ [7.33, 7.45]). At the lower end were “Critical Thinkers” (M = 6.89, 95% CI_CM_ [6.82, 6.96]), “Evidence Based Practice” (M = 6.86, 95% CI_CM_ [6.79, 6.92]), and “Researchers” (M = 6.84, 95% CI_CM_ [6.77, 6.91]). Notably, the Cousineau-Morey CIs for all top-11 items did not overlap those for these three lowest items, indicating higher within-person endorsement of the former group. Full item-level statistics are visualized in Fig. 1.

#### Patient-centered care and compassion

Mauchly’s test indicated a violation of sphericity, W = 0.72, *p* < 0.001. Hence, Greenhouse-Geisser corrections were applied ($\:\epsilon\:$ = 0.92). The repeated measures ANOVA revealed that items differed in their mean scores, F(7.39, 7016.51) = 16.33, *p* < 0.001. Partial eta-squared ($\:{{\eta\:}_{p}}^{2})$ was 0.02 (95% CI [0.01, 0.02]), a small effect. The minimum detectable effect was $\:{{\eta\:}_{p}}^{2}$ = 0.001.

Item means ranged from M = 6.79 (95% CI_CM_ [6.72, 6.86]) to M = 7.43 (95% CI_CM_ [7.37, 7.48]). Eight of 9 items (89%) had point estimates $\:\ge\:$ 7.00. “Caring/Compassionate” was the top-rated item (M = 7.43, 95% CI_CM_ [7.37, 7.48]). Its Cousineau-Morey CI did not overlap those for “Trusted” (M = 7.31, 95% CI_CM_ [7.25, 7.36]) or “Empathetic” (M = 7.22, 95% CI_CM_ [7.16, 7.28]), indicating that these within-person mean differences are unlikely to be zero. Visual inspection further suggests comparatively higher endorsement of the three most strongly rated items relative to the three lowest-rated items: “Talented” (M = 6.79, 95% CI_CM_ [6.72, 6.86]), “Advocates” (M = 7.02, 95% CI_CM_ [6.96, 7.08]), and “Nurturing/Mothering” (M = 7.05, 95% CI_CM_ [6.99, 7.11]). Difference-adjusted Cousineau-Morey CIs did not overlap between these groups.

#### Leadership and influence

Mauchly’s test indicated that sphericity was violated, W = 0.87, *p* < 0.001, therefore Greenhouse-Geisser corrections were applied ($\:\epsilon\:$ = 0.96). The repeated measures ANOVA indicated that item means differed, F(6.74, 6392.97) = 7.65, *p* < 0.001. The effect was very small to small, $\:{{\eta\:}_{p}}^{2}$ = 0.008, 95% CI [0.004, 0.012]. The minimum detectable effect was $\:{{\eta\:}_{p}}^{2}$ = 0.001.

Item means were generally lower than in the preceding subscales, with no items $\:\ge\:$ 7.00 (range M = 6.42 to 6.78). The three top-rated items were “Health Experts” (M = 6.78, 95% CI_CM_ [6.71, 6.86]), “Valued by Society/Healthcare” (M = 6.78, 95% CI_CM_ [6.70, 6.86]), and “Autonomous” (M = 6.73, 95% CI_CM_ [6.66, 6.81]). Their Cousineau-Morey CIs did not overlap those of the lower-rated items (e.g., “Influential”, “Powerful/Decision Makers”, “Leaders”), indicating higher within-person endorsement for the former.

#### Professional identity challenges

Mauchly’s test did not indicate a violation of sphericity, W = 1.00, *p* = 0.62. The uncorrected ANOVA is therefore reported, showing that item means differed, F(2, 1898) = 11.78, *p* < 0.001. The effect was very small to small, $\:{{\eta\:}_{p}}^{2}$ = 0.012, 95% CI [0.004, 0.023]. The minimum detectable effect was $\:{{\eta\:}_{p}}^{2}$ = 0.002.

Item means lay in the upper portion of the middle tercile on the 1–10 scale, ranging from M = 6.06 to 6.44. Cousineau-Morey CIs for “Hard to identify from others” (M = 6.44, 95% CI_CM_ [6.36, 6.51]) and “Subservient” (M = 6.29, 95% CI_CM_ [6.22, 6.37]) did not overlap the CI for the lowest-rated item, “Attire does not reflect professionalism” (M = 6.06, 95% CI_CM_ [5.98, 6.14]), indicating higher within-person endorsement for the former two.

In the NBIS-P-G validation [72], the item “female” loaded on this subscale but was excluded due to a weak association. Since recent reviews [14, 16] mentioned that nursing remains gender stereotyped, we report it post hoc as supplementary information (not part of the validated scale). Its mean was M = 6.78, 95% CI_CM_ [6.70, 6.87], near the upper bound of the middle tercile. Its Cousineau-Morey CI did not overlap those of the *Professional Identity Challenges* items, indicating comparatively higher endorsement.

### Group comparisons of subscale scores

Table 3 shows the group comparisons across demographic (gender, age, education, region), socio-cultural (lifestyles), and psychological (personality metatraits) grouping variables.

#### Gender

Welch’s t-tests with Benjamini-Hochberg correction revealed no significant gender differences for any subscale (all p_BH_$\:\ge\:$ 0.133; Table 3). Effect sizes were very small to small: *Professional Competence and Expertise* d = 0.118, 95% CI [-0.010, 0.246], *Patient-Centered Care and Compassion* d = 0.139, 95% CI [0.011, 0.266], *Leadership and Influence* d = 0.075, 95% CI [-0.052, 0.203], and *Professional Identity Challenges* d = 0.042, 95% CI [-0.085, 0.169]. The minimum detectable effect was d = 0.22.

**Table 3:** Results of group comparisons

		N	Professional Competence and Expertise		Patient-Centered Care and Compassion		Leadership and Influence		Professional Identity Challenges	
			M	SD	M	SD	M	SD	M	SD
Gender	Female	491	7.27	1.50	7.25	1.67	6.66	1.57	6.30	1.98
	Male	459	7.08	1.64	7.01	1.79	6.54	1.70	6.22	1.95
	t, p_BH_, d [95% CI]		1.81, 0.140, 0.118 [-0.010, 0.246]		2.13, 0.133, 0.139 [0.011, 0.266]		1.15, 0.331, 0.075 [-0.052, 0.203]		0.64, 0.519, 0.042 [-0.085, 0.169]	
Age (years)	16–29	243	7.01	1.36	6.96	1.59	6.74	1.35	6.36	1.87
	30–39	217	7.20	1.54	7.10	1.74	6.73	1.65	6.46	1.90
	40–49	192	7.16	1.56	7.17	1.74	6.53	1.74	6.23	1.97
	50–65	298	7.31	1.75	7.27	1.82	6.44	1.76	6.06	2.06
	F, p_BH_, $\:{{\eta\:}_{p}}^{2}$ [95% CI]		1.70, 0.204, 0.010 [0.000, 0.029]		1.54, 0.204, 0.009 [0.000, 0.027]		2.18, 0.204, 0.013 [0.000, 0.034]		1.90, 0.204, 0.011 [0.000, 0.031]	
Education level	Low	218	6.92	1.64	6.83	1.83	6.49	1.71	6.25	2.03
	Mid	321	7.23	1.52	7.19	1.67	6.66	1.54	6.34	1.88
	High	411	7.27	1.56	7.25	1.71	6.61	1.68	6.21	1.99
	F, p_BH_, $\:{{\eta\:}_{p}}^{2}$ [95% CI]		3.65, 0.053, 0.013 [0.000, 0.037]		4.19, 0.053, 0.015 [0.001, 0.040]		0.74, 0.637, 0.003 [0.000, 0.015]		0.43, 0.651, 0.002 [0.000, 0.012]	
Region	North	155	6.97	1.54	7.01_aβ_	1.66	6.42	1.57	6.12	1.85
	West	368	7.32	1.41	7.37_a_	1.58	6.74	1.56	6.47	1.87
	East	193	7.13	1.78	6.94_β_	1.90	6.52	1.76	6.17	2.02
	South	234	7.12	1.64	7.00_β_	1.82	6.57	1.69	6.11	2.11
	F, p_BH_, $\:{{\eta\:}_{p}}^{2}$ [95% CI]		2.25, 0.109, 0.016 [0.000, 0.041]		4.10, 0.028, 0.028 [0.002, 0.060]		1.73, 0.159, 0.012 [0.000, 0.034]		2.32, 0.109, 0.016 [0.000, 0.040]	
Lifestyle	Social Elite	349	7.37_a_	1.54	7.36_a_	1.71	6.98_a_	1.62	6.67_a_	1.92
	Traditionalists	81	7.04_a__β_	1.67	6.78_a__β_	2.03	5.65_β_	1.73	5.16_β_	1.92
	Middle Class	277	6.93_β_	1.55	6.88_β_	1.61	6.35_c_	1.41	6.03_c_	1.78
	Avantgarde	168	7.33_aβ_	1.58	7.33_aβ_	1.78	6.88_a_	1.70	6.42_ac_	2.15
	Underprivileged-Modernized	75	6.98_aβ_	1.54	6.95_aβ_	1.65	6.20_βc_	1.63	6.06_ac_	1.86
	F, p_BH_, $\:{{\eta\:}_{p}}^{2}$ [95% CI]		4.03, 0.003, 0.055 [0.007, 0.105]		4.49, 0.002, 0.062 [0.010, 0.114]		15.23, < 0.001, 0.183 [0.100, 0.257]		11.99, < 0.001, 0.149 [0.072, 0.219]	
Personality type	High S – High P	139	7.81_a_	1.46	7.69_a_	1.58	6.81	1.80	6.20	2.21
	High S – Low P	50	7.07_aβ_	1.68	7.12_aβ_	1.93	6.39	1.70	6.14	1.87
	Low S – High P	54	6.88_β_	1.62	6.67_β_	1.82	6.25	1.52	5.86	1.75
	Low S – Low P	106	6.96_β_	1.63	6.97_β_	1.88	6.30	1.67	6.05	1.89
	Others	601	7.10_β_	1.54	7.07_β_	1.69	6.66	1.59	6.36	1.94
	F, p_BH_ $\:{{\eta\:}_{p}}^{2}$ [95% CI]		7.60, < 0.001, 0.156 [0.055, 0.246]		5.49, < 0.001, 0.118 [0.028, 0.202]		2.37, 0.073, 0.054 [0.000, 0.116]		1.48, 0.211, 0.034 [0.000, 0.084]	

Note: M = Mean; SD = Standard Deviation; CI = Confidence Interval; S = Stability; P = Plasticity. Statistics rows show test statistic, Bejamini-Hochberg adjusted p-value (pBH), and effect size [95% CI]. For Gender: t-value and Cohen’s d. For other variables: F-value and partial eta-squared ($\:{{\eta\:}_{p}}^{2}$). CIs are unadjusted for multiple comparisons. Means with different subscripts within the same column and grouping variable differed significantly (Games-Howell, *p* < 0.05). Detailed pairwise statistics are reported in Table 4

#### Age

Welch’s one-way ANOVAs with Benjamini-Hochberg correction revealed no significant age-group differences (all p_BH_ = 0.204; Table 3). Effects were small at most ($\:{{\eta\:}_{p}}^{2}$ = 0.009-0.013, 95% CI [0.000, 0.027] to [0.000, 0.034]). The minimum detectable effect was $\:{{\eta\:}_{p}}^{2}$ = 0.016.

#### Education

Welch’s one-way ANOVAs with Benjamini-Hochberg correction revealed no significant education-group differences (Table 3). However, *Professional Competence and Expertise* ($$\:{{\eta\:}_{p}}^{2}$$ = 0.013, 95% CI [0.000, 0.037]) and *Patient-Centered Care and Compassion* ($$\:{{\eta\:}_{p}}^{2}$$ = 0.015, 95% CI [0.001, 0.040]) approached significance (both p_BH_ = 0.053). Effects for the other two subscales were negligible ($$\:{{\eta\:}_{p}}^{2}$$$$\:\le\:$$ 0.003). The minimum detectable effect was $$\:{{\eta\:}_{p}}^{2}$$ = 0.014.

#### Region

Welch’s one-way ANOVAs with Benjamini-Hochberg correction revealed one significant regional difference: *Patient-Centered Care and Compassion*, F(3, 432.16) = 4.10, p_BH_ = 0.028, $\:{{\eta\:}_{p}}^{2}$ = 0.028, 95% CI [0.002, 0.060]. Considering the CI, the effect was very small to small with the upper CI bound approaching the medium benchmark.

Games-Howell post-hoc tests showed that western Germany rated this dimension significantly higher than eastern and southern Germany; Cohen’s d CIs indicated very small to small effects (Table 4). Other comparisons were not significant.

The remaining three subscales showed no significant regional effects (p_BH_ = 0.109-0.159; Table 3). Effect sizes were small at most ($\:{{\eta\:}_{p}}^{2}$ = 0.012-0.016, 95% CI [0.000, 0.034] to [0.000, 0.041]).

The minimum detectable effect for the ANOVAs was $\:{{\eta\:}_{p}}^{2}$ = 0.016.

**Table 4:** Significant post-hoc pairwise comparisons

Grouping variable	Subscale	Contrast	$\:\boldsymbol{\Delta\:}$M	*p*	d	95% CI (d)
Region	PCCC	West vs. East	0.43	0.035	0.248	[0.068, 0.428]
		West vs. South	0.37	0.049	0.219	[0.052, 0.386]
Lifestyle	PCE	Social Elite vs. Middle Class	0.44	0.003	0.289	[0.130, 0.447]
	PCCC	Social Elite vs. Middle Class	0.48	0.004	0.286	[0.128, 0.444]
	LI	Social Elite vs. Traditionalists	1.33	< 0.001	0.794	[0.525, 1.060]
		Social Elite vs. Middle Class	0.63	< 0.001	0.416	[0.258, 0.574]
		Social Elite vs. Underprivileged-Modernized	0.77	0.003	0.475	[0.217, 0.732]
		Traditionalists vs. Middle Class	− 0.70	0.010	− 0.443	[-0.709, − 0.175]
		Traditionalists vs. Avantgarde	− 1.23	< 0.001	− 0.719	[-0.995, − 0.440]
		Middle Class vs. Avantgarde	− 0.53	0.006	− 0.342	[-0.539, − 0.144]
		Avantgarde vs. Underprivileged-Modernized	0.67	0.031	0.405	[0.130, 0.678]
	PIC	Social Elite vs. Traditionalists	1.50	< 0.001	0.783	[0.529, 1.043]
		Social Elite vs. Middle Class	0.64	< 0.001	0.343	[0.185, 0.501]
		Traditionalists vs. Middle Class	− 0.87	0.004	− 0.469	[-0.728, − 0.209]
		Traditionalists vs. Avantgarde	−1.25	< 0.001	− 0.614	[-0.881, − 0.346]
		Traditionalists vs. Underprivileged-Modernized	− 0.89	0.030	− 0.472	[-0.790, − 0.153]
Personality type	PCE	High S – High P vs. Low S – High P	0.93	0.004	0.598	[0.263, 0.930]
		High S – High P vs. Low S – Low P	0.85	< 0.001	0.543	[0.283, 0.803]
		High S – High P vs. Others	0.71	< 0.001	0.469	[0.281, 0.655]
	PCCC	High S – High P vs. Low S – High P	1.02	0.004	0.598	[0.261, 0.932]
		High S – High P vs. Low S – Low P	0.72	0.014	0.416	[0.157, 0.674]
		High S – High P vs. Others	0.62	< 0.001	0.377	[0.193, 0.561]

Note: Only statistically significant Games-Howell post-hoc comparisons (*p* < 0.05) are displayed. Contrasts are ordered following the sequence of groups in Table 3. Positive values indicate higher scores for the first-listed group. ΔM = Mean difference; d = Cohen’s d; CI = Confidence Interval; PCE = Professional Competence and Expertise; PCCC = Patient-Centered Care and Compassion; LI = Leadership and Influence; PIC = Professional Identity Challenges; S = Stability; P = Plasticity

#### Lifestyles

Welch’s one-way ANOVAs with Benjamini-Hochberg correction revealed significant lifestyle-group differences for all four subscales (Table 3).

For *Professional Competence and Expertise* (F(4, 274.45) = 4.03, p_BH_ = 0.003, $\:{{\eta\:}_{p}}^{2}$ = 0.055, 95% CI [0.007, 0.105]) and *Patient-Centered Care and Compassion* (F(4, 272.49) = 4.49, p_BH_ = 0.002, $\:{{\eta\:}_{p}}^{2}$ = 0.062, 95% CI [0.010, 0.114]), effects were very small to medium. Both subscales showed the same pattern: the Social Elite rated these dimensions significantly higher than the Middle Class (Table 4). Other comparisons were not significant.

For *Leadership and Influence*, F(4, 271.66) = 15.23, p_BH_ < 0.001, $\:{{\eta\:}_{p}}^{2}$ = 0.183, 95% CI [0.100, 0.257]. The effect was medium to large. The Social Elite and Avantgarde rated nursing leadership highest, while Traditionalists rated it lowest. Both former groups significantly exceeded Traditionalists, Middle Class, and Underprivileged-Modernized groups; the Middle Class also rated leadership higher than Traditionalists (Table 4).

For *Professional Identity Challenges*, F(4, 274.93) = 11.99, p_BH_ < 0.001, $\:{{\eta\:}_{p}}^{2}$ = 0.149, 95% CI [0.072, 0.219]. The effect was medium to large. The Social Elite and Avantgarde perceived the most challenges, Traditionalists the fewest. The Social Elite perceived significantly more challenges than Traditionalists and Middle Class; Traditionalists perceived significantly fewer challenges than Middle Class, Avantgarde, and Underprivileged-Modernized groups (Table 4).

The minimum detectable effect for the ANOVAs was $\:{{\eta\:}_{p}}^{2}$ = 0.017.

#### Personality metatraits

Welch’s one-way ANOVAs with Benjamini-Hochberg correction revealed significant personality segment differences for two subscales (Table 3).

For *Professional Competence and Expertise* (F(4, 164.63) = 7.60, p_BH_ < 0.001, $\:{{\eta\:}_{p}}^{2}$ = 0.156, 95% CI [0.055, 0.246]) and *Patient-Centered Care and Compassion* (F(4, 163.82) = 5.49, p_BH_ < 0.001, $\:{{\eta\:}_{p}}^{2}$ = 0.118, 95% CI [0.028, 0.202]), effects were small to large. Both subscales showed the same pattern: the High Stability-High Plasticity segment rated these dimensions significantly higher than Low Stability-High Plasticity, Low Stability-Low Plasticity, and Others segments (Table 4). Other comparisons were not significant.

No significant effects emerged for *Leadership and Influence* (p_BH_ = 0.073, $\:{{\eta\:}_{p}}^{2}$ = 0.054, 95% CI [0.000, 0.116]) or *Professional Identity Challenges* (p_BH_ = 0.211, $\:{{\eta\:}_{p}}^{2}$ = 0.034, 95% CI [0.000, 0.084]).

The minimum detectable effect for the ANOVAs was $\:{{\eta\:}_{p}}^{2}$ = 0.017.

To assess whether the results for *Professional Competence and Expertise* and *Patient-Centered Care and Compassion* were robust beyond the categorical segmentation, we conducted post hoc multiple linear regressions using continuous metatrait scores, employing the HC3 heteroscedasticity-consistent estimator for robust standard error estimation. The two subscales were regressed on Stability, Plasticity, and their interaction, controlling for demographics. Higher Stability predicted more favorable perceptions (B = 0.11 / 0.13, both *p* <0.001; unstandardized coefficients based on metatrait composites derived from z-standardized trait scores), while Plasticity showed no main effects (both *p* > 0.05). Positive Stability × Plasticity interactions emerged for both subscales (B = 0.05, *p* = 0.001 / 0.007). Greater Stability, particularly when combined with higher Plasticity, was associated with more positive nursing brand image perceptions.

## Discussion

This study provides the first population-level assessment of nursing’s brand image in the German public using a validated instrument, and examines its variation across sociodemographic, lifestyle, and personality characteristics. The following discussion interprets the findings within the brand image framework that guided this study, drawing on complementary interpretive lenses from sociology and psychology where they illuminate dimension- or segment-specific results.

### Core brand image dimensions in the German public

The brand image was characterized primarily by associations of *Professional Competence and Expertise* and *Patient-Centered Care and Compassion*, with no significant difference between these subscales’ mean scores. This dual prominence of competence and relational associations aligns with the Stereotype Content Model [113] and other social evaluation theories [114], which have become established interpretative frameworks in branding research [115] and identify “competence” (perceived capability and efficacy) and “warmth” (perceived kindness and relational orientation) as core dimensions of social and brand perception [115].

That both dimensions are equally endorsed in the German public is noteworthy. Prior German evidence suggested social qualities substantially outweigh competence associations among adolescents [61], and media analyses indicated limited emphasis on the profession’s capabilities [48] or even portrayals of nurses as lacking competencies [60]. Our finding also contrasts the hypothesis that nursing in Germany is generally subject to “low competence attribution” [116]. Additionally, it contrasts with international research emphasizing that public perception prioritizes caring and empathy, with warmth attributes (friendliness, compassion) outweighing competence markers [24–26]. However, it converges with Zhou et al.’s [26] findings for the United States and China, where “caregiver attributes” (skilled, professional, caring/compassionate) and “collaborative/communicator” qualities (empathetic, competent) were most strongly endorsed.

The robust presence of both competence and warmth associations may have positive implications, as perceived warmth and competence of health professionals have been linked to favorable health-related outcomes [117]. Whether such effects extend to nursing’s brand image at the profession level, however, warrants investigation.

Notably, the NBIS-P-G validation study [72] found a positive correlation between *Professional Competence and Expertise* and *Patient-Centered Care and Compassion*, suggesting a non-ambivalent interaction rather than the warmth-competence trade-off observed in other contexts [115]. Whether one dimension serves as a prerequisite for attributions on the other (primacy) [114, 115] – with implications for strategic sequencing of branding messages – merits future investigation.

Nevertheless, while competence and warmth represent core assets of nursing’s brand image, the observed endorsement levels (M $\:\sim$ 7.1–7.2, upper tercile on a 10-point scale) were favorable but not very high, suggesting room for enhancement given nursing image’s importance for workforce and other outcomes.

Item-level patterns provide additional nuance to these overall findings. Within *Professional Competence and Expertise*, associations of nurses as “Critical Thinkers” (M = 6.89), “Researchers” (M = 6.84), and with “Evidence Based Practice” (M = 6.86) were comparatively less endorsed. This aligns with Labonte’s [48] observation that nursing’s image in Germany remains shaped by historical remnants, and is consistent with Germany’s low academization rate, which falls substantially below recommended minimum levels [118]. Against this background, aspects related to academic qualification, specialized expertise, and scientific foundations of nursing may be less salient in public perception. Within *Patient-Centered Care and Compassion*, “Advocates” (M = 7.02), e.g., received relatively lower endorsement, suggesting that nursing’s patient advocacy role remains less prominent in public awareness.

To further strengthen these perceptions, both general and domain-specific approaches warrant consideration. At a general level, competence communication strategies could enable nurses to articulate their technical and relational capabilities more effectively [116, 119]. At a domain-specific level, advancing nursing academization [118] may contribute to strengthening public associations with critical thinking, research, and evidence-based practice. Reinforcing advocacy perceptions could be supported by broadening the scope and visibility of nurses’ advocacy roles [120, 121]. Additionally, occupational branding campaigns [122] could make a valuable contribution by strategically representing nursing work as evidence-based and highlighting nurses’ roles in research and advocacy. Such campaigns could showcase nursing research contributions, evidence-based care innovations, or use narrative storytelling to raise public perception of these less salient attributes.

### Leadership and professional identity perceptions

*Leadership and Influence* received significantly lower ratings (M = 6.60, middle tercile) than *Professional Competence and Expertise* or *Patient-Centered Care and Compassion*. This pattern aligns with international research finding that nursing leadership associations are less prominently endorsed [24–26]. It also reflects broader evidence that nursing is viewed as having limited autonomy and that the public often lacks understanding of nursing’s role diversity across management, education, research, and policy domains [14, 16].

Item-level analysis revealed variation within this subscale: while “Health Experts” (M = 6.78) and “Valued by Society/Healthcare” (M = 6.78) received moderate endorsement, attributes directly indicating decisional authority – “Influential” (M = 6.57), “Powerful / Decision Makers” (M = 6.48), and “Leaders” (M = 6.42) – were rated lower. This pattern suggests that while endorsement of nurses’ expertise and societal value is relatively strong, it may not readily extend to perceptions of legitimate authority in healthcare governance and decision-making.

To interpret these brand association differences, Bourdieu’s field and capital theory, which has informed both branding research [123] and nursing scholarship [124–126] and also anchors the lifestyle typology used in this study, offers a useful lens. While nursing’s cultural capital (professional and relational competencies) and social capital (compassionate relationships) appear recognized, these forms of capital seem to be accorded comparatively limited symbolic value in matters of leadership and influence within the healthcare field.

The comparatively weaker leadership perceptions may in part reflect structural features of the German healthcare system that may limit public visibility of nursing leadership in research (e.g., low academization rates, limited university infrastructure [118]), clinical practice (e.g., underdeveloped leadership roles [118]), and health policy (e.g., absence of institutionalized self-governance structures in most German states [127]).

Recent research [16, 128–130] has proposed strategies for enhancing leadership and influence perceptions, including positioning nursing as a leadership profession [129], developing nurse ambassadors in visible leadership roles, increasing public visibility through social media [128, 130], and implementing empowerment initiatives through supportive work environments and systematic development of “influence skills” [130, 131]. In the German context, such initiatives could benefit from being accompanied by structural measures addressing the academization, clinical leadership, and professional governance gaps noted above.

*Professional Identity Challenges* requires inverse interpretation: higher values reflect perceptions of subordination and lacking differentiation from other health professions. With a mean of M = 6.26 – lower than positively valenced subscales but clearly above the scale midpoint – the data suggest persistent image challenges regarding professional status and distinctiveness. This mirrors Zhou et al.’s [26] findings of perceived subservience and limited differentiation in the USA and particularly China, and aligns with international reviews indicating nursing continues to be viewed as low-status and subordinate, with visual cues inadequately differentiating nurses from other staff [14, 16].

While the “Subservient” item (M = 6.29) reflects the previously discussed limited perceptions of nursing leadership, the attire-related items “Hard to identify from others” (M = 6.44) and “Attire does not reflect professionalism” (M = 6.06) warrant separate consideration as markers of nursing’s extrinsic image dimension [14].

Research on person perception suggests that dress serves as a cue for inferring social category membership and professional status [132]. Our findings indicate that nursing attire in Germany may inadequately serve these functions, with the public perceiving limited role differentiation and professional signaling, consistent with international evidence that nursing attire affects perceived trust, professionalism, and role clarity [133, 134]. Strategies such as differentiating uniforms to signal professional boundaries and advanced roles [133] or using aspirational aesthetics to increase perceived status [135] merit consideration, though attire design requires contextual sensitivity across care settings and organizational contexts [136–138]. In Germany, nursing attire is determined at organizational level within occupational safety and infection control regulations, an approach that allows context-sensitive design but may constrain consistent professional signaling across the profession.

Relatedly, gender associations remain part of nursing’s image challenges. Although the item “female” was excluded from the validated NBIS-P-G due to weak factor association [72], its endorsement (M = 6.78, upper bound of middle tercile) warrants brief consideration given international evidence that nursing remains viewed as a feminine profession shaped by gender stereotypes [14, 16]. This endorsement reflects nursing’s persistent feminization in German public perception, mirroring workforce demographics (approximately 80% female [53]) while rooted in cultural-historical associations between gender and care work [139]. Shifting these perceptions may require fundamental approaches from decoupling care work from femininity to equitable remuneration and enhanced career pathway visibility [139].

Ultimately, addressing the limitations across all four brand image dimensions documented here may benefit not only from incremental measures but also from broader transformation of the German healthcare system. Initiatives such as the Robert Bosch Stiftung’s “Restart!” envision such transformation: a shift from physician-centered, disease focused-structures toward a patient-centered, multi-professional system grounded in health promotion, collaboration at eye level, and highest qualification standards across professions [140]. Such changes could, over time, contribute to redefining nursing’s position within healthcare and its public image.

### Sociodemographic variation in brand perceptions

Our analyses revealed no statistically significant differences in nursing brand image perceptions across sociodemographic groups for any of the four dimensions, with one exception: a small regional difference in *Patient-Centered Care and Compassion* (western Germany rated this dimension higher than eastern and southern Germany). Following Jacob et al.’s framework for interpreting null results [141], we consider not only statistical significance but also whether estimated effects are substantively small and measured with precision.

For *gender*, effect size point estimates were very small for *Professional Competence and Expertise* and *Patient-Centered Care and Compassion*, and negligible for *Leadership and Influence* and *Professional Identity Challenges*. However, confidence intervals were wide, with upper bounds extending into the small effect range for the first three subscales, indicating our data remain compatible with small gender differences, with women potentially perceiving these dimensions slightly more positively. Prior international findings are mixed, with some studies reporting no differences [24, 26] and others finding men less likely to hold positive nursing images [39]; our findings suggest any such differences in Germany are subtle at most.

For *age*, effect size point estimates ranged from very small to small, with wide confidence intervals indicating compatibility with small age-related differences. International studies show mixed results: some report no age effects [26], others suggest older adults attribute higher prestige to nursing [24] or hold more traditional views [43, 44]. Our pattern – older individuals potentially rating competence and care higher, leadership and identity challenges lower – could partially reflect the latter: higher competence/care ratings might indicate prestige attributions, while lower leadership and identity challenge perceptions could mirror traditional views. However, given imprecise estimates and lack of significance, our findings primarily align with no-effects research [26].

For *education*, effect size point estimates were small for *Professional Competence and Expertise* and *Patient-Centered Care and Compassion* and negligible for the other two subscales. Wide confidence intervals with upper bounds in the small effect region indicate compatibility with small education-related differences. While one international study found educational attainment positively correlated with nursing perceptions [26], our imprecise, non-significant estimates preclude conclusions about education-related differences in the German context.

For *region*, beyond the significant *Patient-Centered Care and Compassion* difference noted above, data remain compatible with small regional effects for other subscales. Given Germany’s federal structure and regional healthcare variation [142], more pronounced differences would have been conceivable. Future research could examine whether regional image variation is more evident at finer geographical resolution (federal states, districts, rural-urban contexts).

In summary, our data do not provide strong evidence for substantive sociodemographic differences but cannot be considered true nulls; estimates were imprecise and remain compatible with small effects. This suggests that basic brand perceptions are relatively similar across demographic segments of the German public. However, this does not imply that image related-initiatives should be demographically uniform: brand positioning objectives may differ across segments, and communication strategies and tactics (including content, channels, and creative execution) require tailoring to demographic groups’ specific ecosystems, media use, and sense-making processes to optimize reach and resonance [143, 144].

### Lifestyle-based variation in brand perceptions

Our results indicate statistically significant differences in image perceptions across lifestyle groups, with effect sizes ranging from very-small-to-medium for *Professional Competence and Expertise* and *Patient-Centered Care and Compassion* to medium-to-large for *Leadership and Influence* and *Professional Identity Challenges*. These findings resonate with the “lifestyle as a cause” perspective [145], which considers lifestyles – structured by economic and cultural capital endowments and value orientations – as regulating instances for perceptions, attitudes and behaviors in various domains [70, 86] including branding [146]. Our results suggest that this regulatory function extends to associative networks regarding nursing.

However, the pattern of differences varied across dimensions. For competence and care, only one significant difference emerged: the Social Elite rated both higher than the Middle Class. This suggests the Stereotype Content Model’s [113] core dimensions – “competence” and “warmth” – are perceived rather uniformly across most lifestyle groups. The Social Elite’s comparatively higher endorsements may relate to their superior economic and cultural capital endowments [70, 86, 147], potentially including greater access to differentiated healthcare and nursing experiences [148] and higher awareness of nursing professionalization through quality media consumption [147, 149].

In contrast, *Leadership and Influence* and *Professional Identity Challenges* showed pronounced differences between lifestyle groups, with the Social Elite and Avantgarde rating both dimensions highest and Traditionalists lowest. From a lifestyle-analytic perspective [70, 86, 147], these differences may reflect the groups’ distinct capital endowments and value orientations. The Social Elite’s and Avantgarde’s professional contexts, which often involve leadership and autonomy as defining action logics [70, 86, 147], may facilitate recognizing these qualities in nursing. The Avantgarde’s egalitarian gender attitudes [86] may further support leadership attribution to a female-dominated profession. Conversely, Traditionalists’ lower perceptions may reflect traditional values [86] that naturalize conventional healthcare hierarchies and media consumption patterns providing less exposure to nursing professionalization discourse [147, 149]. While these interpretations are plausible, the specific mechanisms require empirical verification in future research.

In summary, lifestyle, as a regulating instance rooted in differential capital endowments and value orientations [86], is associated with variation in nursing’s brand image. These results highlight the importance of nursing image research moving beyond sociodemographic variables to adequately capture heterogeneity in brand perceptions and support the design of targeted, group-specific image interventions.

### Personality-based variation in brand perceptions

We identified significant personality-segment differences for *Professional Competence and Expertise* and *Patient-Centered Care and Compassion*. Effect sizes were moderate to large by point estimate ($\:{{\eta\:}_{p}}^{2}$ = 0.156 and 0.118), though wide confidence intervals ([0.055, 0.246] and [0.028, 0.202]) indicate considerable uncertainty about the precise magnitude, with the data compatible with effects ranging from small to large. The High Stability-High Plasticity segment showed the strongest endorsements, rating both dimensions significantly higher than Low Stability-High Plasticity, Low Stability-Low Plasticity, and Others segments, while not differing significantly from the High Stability-Low Plasticity segment. This pattern underscores the relevance of Stability, which is corroborated by the post hoc regressions: higher Stability was associated with more favorable perceptions, whereas Plasticity exhibited no main effects. Positive Stability × Plasticity interactions indicated that Plasticity amplifies the extent to which a stable personality profile leads to stronger competence and care perceptions. These findings extend brand research showing that personality traits affect brand perceptions [150–152] to nursing’s brand image.

To understand why higher Stability is associated with more favorable brand perceptions, Cognitive-Adaptive Trait Theory (CATT) from personality psychology may provide a useful interpretative lens. CATT posits that traits reflect alternative adaptive strategies to the environment, associated with specific cognitive biases in perception, attention, and memory [153]. Stability, characterized by high conscientiousness and agreeableness and low neuroticism [154], may predispose individuals toward greater sensitivity to warmth and prosocial cues (agreeableness), heightened attention to sustained professional effort (conscientiousness), and more favorable appraisal of professional competence (lower neuroticism). These cognitive tendencies make stronger competence and care associations theoretically plausible. Plasticity may amplify these perceptions when combined with Stability, as suggested by the positive interaction effect. Extraversion may facilitate processing of positive social signals, while openness may enhance receptivity to complex forms of competence.

Personality-segment differences did not emerge for *Leadership and Influence* or *Professional Identity Challenges*, despite theoretical considerations that Plasticity – through openness to ideas challenging established healthcare structures and extraversion-related agency [155] – might facilitate stronger endorsement of these dimensions. Why these effects did not emerge warrants further investigation.

Although personality-related differences were limited to competence and care perceptions and effect magnitudes carry considerable uncertainty, these findings nonetheless suggest personality represents an additional relevant segmentation dimension. Whether personality-fitted nursing brand image initiatives – an approach increasingly explored in other domains [156, 157] – can effectively enhance nursing’s brand image merits systematic investigation, particularly given this dimension-specificity.

### Limitations and paths for future research

While this study offers valuable insights into the brand image of nursing in the German public and its heterogeneity, several limitations must be acknowledged.

First, there are limitations inherent in the study design. The present study relied on the dataset previously used to validate the NBIS-P-G. Thus, the instrument’s measurement properties were not cross-validated in an independent sample, warranting replication. Additionally, the cross-sectional design only permits a snapshot assessment of nursing’s brand image, precluding analysis of potential changes over time or causal inference regarding antecedents.

Second, sampling-related limitations may affect external validity. Although a large quota-based sample approximating national distributions of age, gender, education, and region supports a degree of generalizability, non-probability online panel sampling introduces potential bias [78, 79, 96]. Economic self-selection may have influenced panel composition [78], though quotas serving as socioeconomic proxies and modest standard incentives were applied to mitigate this. The exclusive use of an online panel excluded individuals without internet access from the frame population, though recent data indicate near-universal internet use (98–100%) in the surveyed age range in Germany [144], suggesting minimal coverage bias from this source. Within the online format, overrepresentation of digitally literate individuals remains possible, which could affect overall brand image levels. Such individuals may have greater exposure to nursing-related online content such as nurse influencer activity or health-related media, potentially influencing their nursing perceptions differently from less digitally engaged individuals. The age range was limited to 16–65 years, excluding younger adolescents and older adults who may hold distinct perceptions. Older adults in particular are among the most intensive users of healthcare services and may have more direct nursing experiences, potentially leading to different brand image perceptions than those found in the surveyed population. Additionally, the binary gender classification required for quota setting precluded examination of perceptions among gender-diverse individuals. Thus, while the quota-based design and sample size support cautious generalization to the German public aged 16–65, inference remains limited given the data are not from a probability sample, warranting replication with additional samples.

Third, measurement limitations must be acknowledged. Because all constructs were assessed via self-report, common method variance cannot be ruled out. Although procedural remedies were implemented [101], the absence of a latent variable framework precluded explicit modeling of method effects. Regarding personality assessment, our operationalization of Stability and Plasticity used composites of z-standardized Big Five traits rather than latent variable modeling. Although this follows established precedent [94], composites conflate shared variance among constituent traits – which theoretically defines the metatraits – with trait-specific variance, potentially attenuating, amplifying, or altering effects. Future research employing latent-factor modeling could provide more construct-valid analyses.

Fourth, analytical limitations warrant consideration. While the study design provided sufficient statistical power to detect medium-to-small effects for sociodemographic comparisons (minimum detectable: d = 0.22 for gender; $\:{{\eta\:}_{p}}^{2}$ = 0.014-0.016 for other demographics), most observed effect size point estimates fell below these thresholds. Consequently, our study had insufficient power to reliably detect the very small effects that our data remain compatible with, limiting conclusions about subtle sociodemographic variation in nursing brand image. While segmenting into discrete personality groups is consistent with applied branding research, where groups serve as a basis for targeting communication, the ± 0.5 SD thresholds used for segmentation were based on convention [95] rather than theoretically or empirically derived. Crossing two dimensions at ± 0.5 SD systematically produces a larger “Others” category and smaller quadrant groups, limiting statistical precision for comparisons involving the latter. Post-hoc regressions using continuous metatrait scores yielded results convergent to the segmentation, suggesting stability of findings, but future analyses using alternative analytical approaches would further strengthen confidence. Additionally, potential interactions between grouping variables were not examined. Personal or family healthcare experiences, which likely influence nursing perceptions, were not captured, precluding differentiation between perceptions based on direct experience versus mediated representations.

Beyond addressing these limitations, future research could pursue several directions. Methodologically, longitudinal designs could track image dynamics and examine effects of policy reforms such as the recent legislation initiative expanding nursing competencies [51]. Qualitative approaches including brand associative networks and metaphor-based elicitation techniques [69] could illuminate deeper nuances of image perceptions. Broader age inclusion would enable examination of perceptions among younger adolescents and older adults. Cross-national comparative studies using harmonized measures would help contextualize German findings internationally. Moreover, Bayesian approaches could complement the frequentist analyses employed in this study. Bayesian analyses provide evidence both for and against the null hypothesis, allowing discrimination between absence of evidence and evidence of absence, and offer continuous rather than dichotomous measures of evidential support [158–160]. Additionally, they enable evaluation of informative hypotheses specifying theory-based orderings among means [159] and help mitigate multiplicity concerns when evaluating multiple hypotheses simultaneously [158].

Substantively, research could explore additional potential sources of image heterogeneity, including ethnicity and migration background (relevant given Germany’s immigration history and nursing workforce internationalization [161]), finer-grained lifestyle typologies [70], political-communicative milieus [162], and extended personality models [154]. Alternative segmentation criteria such as health literacy, healthcare needs [163], or insurance status merit investigation, as do latent profile analyses identifying latent subpopulations with distinct brand perceptions. Research examining mediating pathways linking lifestyle and personality to image perceptions would advance understanding of the mechanisms of brand image emergence.

From an applied perspective, intervention studies testing segment-targeted communication strategies would bridge descriptive findings and practical action. Examination of brand images for nursing subgroups such as clinical, geriatric, or pediatric nursing could reveal setting-specific perception patterns. Research examining how nursing’s public brand image relates to outcomes such as recruitment, retention, and care quality within the German context would contribute to further clarifying the practical implications of the perceptions documented here.

## Conclusions

This study provides the first population-level assessment of nursing’s brand image in the German public using a validated instrument (NBIS-P-G). Nursing’s brand image is anchored in two core assets – professional competence/expertise and patient-centered care/compassion – endorsed at comparable, moderately favorable levels. Item-level analyses show, however, that even within these dimensions, facets reflecting nursing’s academic and professional scope are less salient: associations with critical thinking, research, evidence-based practice, and advocacy were comparatively weaker. Leadership and influence were rated lower, and professional identity challenges (subservience, limited distinctiveness) were clearly endorsed, indicating limitations in perceived authority, autonomy, and role clarity. While sociodemographic differences were minimal, lifestyle and personality characteristics emerged as meaningful sources of brand image heterogeneity.

For practice, these results suggest brand management priorities at two levels to build and strengthen nursing’s brand image. Regarding brand positioning, stakeholders involved in nursing’s brand image cocreation may focus on preserving and further strengthening the brand’s established core assets of professional competence/expertise and patient-centered care/compassion. Particular attention could be given to emphasizing nursing’s evolving academic and professional scope. In parallel, efforts could focus on systematically broadening the brand’s association base to include leadership and influence in research, practice, and policy. Additionally, co-positioning the brand more clearly, distinctively, and authoritatively within the broader health professions brand ecosystem could help address professional identity challenges. The brand positioning could guide segment-specific brand execution, including communication accounting for lifestyle and personality variation, and brand narratives that enhance leadership visibility, role differentiation, and professional signaling. Achieving these priorities involves multiple stakeholders in nursing’s brand image cocreation [129], including individual nurses, professional associations, nursing education institutions, policymakers, and media and communication practitioners. Overall, the study’s findings offer entry points for each of these stakeholder groups, provide an empirical foundation for strengthening nursing’s brand image in Germany and establish a baseline for evaluating future branding initiatives.

## Supplementary Information

Below is the link to the electronic supplementary material.

Supplementary Material 1

Supplementary Material 2

Supplementary Material 3

## Data Availability

The datasets used and/or analyzed during the current study are available from the corresponding author on reasonable request.
