# Supplementary material for: Competent and compassionate, but not leading? A cross-sectional study of nursing’s brand image in the German public
Source: BMC Nurs. 2026 Apr 28;25:481. doi: 10.1186/s12912-026-04683-z (PMC13214203; doi:10.1186/s12912-026-04683-z)
Supplement: Supplementary file 1 — Supplementary Material 1 [file 12912_2026_4683_MOESM1_ESM.docx]

**Table.** NBIS-P-G items and correspondence to original NBIS (English)

| **NBIS-P-G items (German)** | | **NBIS (English)** |
| --- | --- | --- |
| ***Subscale 1: Professional Competence and Expertise*** | |  |
| 1 | fachkundig | Skilled |
| 2 | professionell | Professional |
| 3 | Brückenbauer*innen in der Kommunikation | Communicators |
| 4 | umfassende Ausbildung | Extensive Training |
| 5 | kompetent | Competent |
| 6 | ganzheitlicher Ansatz | Holistic Approach |
| 7 | interprofessionell | Interprofessional |
| 8 | ehrlich/Integrität | Honest/Integrity |
| 9 | ethisch | Ethical |
| 10 | achtsam und umsichtig | Intuitive/Thoughtful |
| 11 | verlässlich/zuverlässig | Reliable/Dependable |
| 12 | evidenzbasierte Praxis | Evidence Based Practice |
| 13 | Pflegewissenschaftler*innen | Researchers |
| 14 | reflektiert/kritisch denkend | Critical Thinkers |
| 15 | sachkundig/intelligent | Knowledgeable/Intelligent |
| 16 | interprofessionelle Partner*innen | Collaborators/Facilitators |
| ***Subscale 2: Patient-Centered Care and Compassion*** | |  |
| 17 | fürsorglich/mitfühlend | Caring/Compassionate |
| 18 | verbringt die meiste Zeit mit Patient*innen | Spends Most Time With Patients |
| 19 | empathisch | Empathetic |
| 20 | genießt Vertrauen | Trusted |
| 21 | patientenorientiert | Patient Centered/Focused |
| 22 | umsorgend/bemutternd | Nurturing/Mothering |
| 23 | Fürsprecher*innen | Advocates |
| 24 | ärztliche Hilfskraft | Physician's Assistant |
| 25 | talentiert/begabt | Talented |
| ***Subscale 3: Leadership and Influence*** | |  |
| 26 | mächtig/Entscheidungsträger*innen | Powerful/Decision Makers |
| 27 | Führungspersonen | Leaders |
| 28 | einflussreich | Influential |
| 29 | höhere Bildungsabschlüsse | Advanced Degrees |
| 30 | von Gesellschaft/Gesundheitswesen wertgeschätzt | Valued by Society/Healthcare |
| 31 | eigenständig | Autonomous |
| 32 | kompetent im Umgang mit Technik | Technological |
| 33 | Gesundheitsexpert*innen | Health Experts |
| ***Subscale 4: Professional Identity Challenges*** | |  |
| 34 | untergeordnet/dienend | Subservient |
| 35 | Berufskleidung vermittelt keine Professionalität | White Cap/Uniform |
| 36 | schwer von anderen zu unterscheiden | Hard to identify from others |

Note: English items are displayed for illustrative purposes only; all items were presented in German.
