# Supplementary material for: Competent and compassionate, but not leading? A cross-sectional study of nursing’s brand image in the German public
Source: BMC Nurs. 2026 Apr 28;25:481. doi: 10.1186/s12912-026-04683-z (PMC13214203; doi:10.1186/s12912-026-04683-z)
Supplement: Supplementary file 2 — Supplementary Material 2 [file 12912_2026_4683_MOESM2_ESM.docx]

**Metatrait derivation details**

***Personality traits and scoring***

The Big Five personality traits were measured with the validated German BFI-10 [1], comprising two items per trait (one reverse-keyed). Items were rated on a 5-point response scale (1 = does not apply at all; 5 = applies completely). Trait scores were computed as the mean of the two items per trait.

***Metatrait derivation***

Following Dunkel et al. [2], metatraits were derived as metric composites from z-standardized trait scores:

- Stability = z(Conscientiousness) + z(Agreeableness) – z(Neuroticism)
- Plasticity = z(Extraversion) + z(Openness)

Stability reflects tendencies to maintain stability and avoid disruption, characterized by high Conscientiousness and Agreeableness and low Neuroticism (or high Emotional Stability). Plasticity reflects tendencies to explore and engage flexibly with novelty, characterized by high Extraversion and Openness [3, 4].

***References***

1. Rammstedt B, Kemper CJ, Klein MC, Beierlein C, Kovaleva A. Big Five Inventory (BFI-10). Zusammenstellung sozialwissenschaftlicher Items und Skalen (ZIS). 2014. https://doi.org/10.6102/ZIS76.

2. Dunkel CS, van der Linden D, de Baca TC, Boutwell BB, Nedelec JL, Petrou P. The Association of Perceived Neighborhood Safety and Inequality with Personality. Evolutionary Psychological Science. 2020;6:354–66. https://doi.org/10.1007/s40806-020-00243-z.

3. DeYoung CG. Higher-order factors of the Big Five in a multi-informant sample. J Pers Soc Psychol. 2006;91:1138–51. https://doi.org/10.1037/0022-3514.91.6.1138.

4. DeYoung CG. Cybernetic Big Five Theory. Journal of Research in Personality. 2015;56 June:33–58. https://doi.org/10.1016/j.jrp.2014.07.004.
