# Supplementary material for: Competent and compassionate, but not leading? A cross-sectional study of nursing’s brand image in the German public
Source: BMC Nurs. 2026 Apr 28;25:481. doi: 10.1186/s12912-026-04683-z (PMC13214203; doi:10.1186/s12912-026-04683-z)
Supplement: Supplementary file 3 — Supplementary Material 3 [file 12912_2026_4683_MOESM3_ESM.docx]

**Effect-size sensitivity analyses**

***Rationale***

Because the sample size for the present study’s analyses was fixed by the NBIS-P-G validation dataset (N = 950), no a priori power analysis was conducted. Instead, effect-size sensitivity analyses were performed to determine the minimum detectable effects for the primary statistical analyses at α = .05 and 80% power. Sensitivity analyses were conducted in G*Power (v3.1). Observed (post hoc) power was not computed, as it is a monotonic transformation of the p-value and provides limited information value.

***Sensitivity analyses for repeated-measures ANOVAs***

For repeated measures ANOVAs examining differences among NBIS-P-G subscale means and item-level endorsement patterns within subscales, sensitivity analyses assumed:

- moderate population intercorrelations among repeated measures (r = .50), consistent with expected halo effects and internal consistency;
- moderate sphericity violations (ε = .75).

Under these assumptions, the minimum detectable partial eta-squared was .002 for subscale comparisons and .001-.002 for item-level within-subscale analyses.

***Sensitivity analyses for paired t-tests***

For post-hoc paired t-tests comparing NBIS-P-G subscales, sensitivity analyses were conducted assuming:

- two-sided testing,
- Bonferroni-adjusted α = .008 (.05 / 6 contrasts), as a conservative approximation to the Benjamini-Hochberg procedure.

The resulting minimum detectable effect size was Cohen’s d_z_ = .113

***Sensitivity analyses for independent-samples t-tests***

For comparing gender groups across subscales, sensitivity analyses were conducted using independent-samples t-test models with:

- two-sided Bonferroni-adjusted α = .013 (.05 / 4 comparisons),
- observed group sizes (female n = 491, male n = 459).

Although G*Power assumes equal variances, this provides a close approximation to Welch’s t-test, which exhibits minimal power loss.

The minimum detectable effect size was Cohen’s d = .22.

***Sensitivity analyses for one-way ANOVAs***

For multi-group comparisons (age, education, region, lifestyle types, personality segments), sensitivity analyses were conducted using standard one-way ANOVA models with:

- Bonferroni-adjusted α = .013 (.05 / 4 omnibus tests per grouping variable),
- group numbers ranging from three to five levels.

G*Power assumes equal variances and group sizes; however, Welch’s ANOVA shows comparable power under typical conditions, making this a reasonable approximation.

The minimum detectable effect size for omnibus tests was partial eta-squared = .014-.017.

Because the omnibus ANOVA addressed the primary inferential question (whether group differences exist), separate sensitivity analyses for conditional Games-Howell post-hoc tests were not performed.
